# Comparison of clinical and imaging features between pulmonary tuberculosis complicated with lung cancer and simple pulmonary tuberculosis: a systematic review and meta-analysis

**DOI:** 10.1017/S0950268822000176

**Published:** 2022-02-02

**Authors:** Weiguo Sun, Lingxia Zhang, Jianqin Liang, Xianan Li, Yimei Yang, Wenna Sun, Jianghou Hou

**Affiliations:** 1 Tuberculosis Prevention and Control Key Laboratory/Beijing Key Laboratory of New Techniques of Tuberculosis Diagnosis and Treatment, Institute for Tuberculosis Research, the 8th Medical Center, Chinese PLA General Hospital, Beijing, China; 2 Kunming City Maternal and Child Health Hospital, Kunming, Yunnan, China

**Keywords:** Clinical symptoms, imaging features, lung cancer, meta-analysis, pulmonary tuberculosis

## Abstract

This review aimed to compare the clinical features and CT imaging features between patients with pulmonary tuberculosis (PTB) and lung cancer and patients with PTB alone. That would help to analyse the differences between the two and consequently providing a theoretical basis for the clinical diagnosis and treatment for the patients. Relevant case-control studies focusing on the clinical and CT imaging characteristics between PTB with lung cancer and PTB alone were systematically searched from five electronic databases. Odds ratios (ORs) and 95% confidence intervals (CIs) were calculated for comparison. As of 2021-07-06, a total of 1735 articles were retrieved. But only 15 articles were finally included for meta-analysis. The results showed a higher proportion of irritable cough, haemorrhagic pleural effusion and lower proportion of night sweating in PTB patients with lung cancer than in PTB patients, and the differences were statistically significant (irritable cough: OR 2.43, 95% CI 1.43–4.11; haemorrhagic pleural effusion: OR 5.73, 95% CI 1.63–20.12; night sweating: OR 0.56, 95% CI 0.36–0.87). In addition, there are many differences in the imaging characteristics of the two types of patients. In conclusion, this review summarises the similarities and differences in clinical symptoms and imaging features between patients with PTB and lung cancer and patients with PTB alone, suggesting that we should be alert to the occurrence of lung cancer in patients with obsolete PTB relapse.

## Introduction

Tuberculosis (TB) is a chronic infectious disease caused by *Mycobacterium tuberculosis*. It often invades multiple organs throughout the body, with pulmonary tuberculosis (PTB) as the most common one [1]. The main respiratory symptoms of PTB are long-term cough and expectoration, accompanied by haemoptysis, chest pain, dyspnoea or others, and its systemic symptoms mainly include low-grade fever, night sweating, fatigue, loss of appetite, emaciation [1]. In recent years, relevant statistics have shown that the incidence of lung cancer gradually tends to be younger, and more males are affected than females. In lung cancer patients, adenocarcinoma is predominant in both male and female patients, while squamous cell carcinoma occupies the majority in patients with smoking and drinking behaviours [2]. Primary lung cancer is characterised by cough, bloody sputum, shortness of breath and emaciation, while tumour metastasis causes hoarseness, dysphagia, chest pain and headache [2]. PTB, it should be noted, is one of the major risk factors for the incidence of lung cancer, and a correlation between the incidence of the two has been proved. Specifically, the incidence of lung cancer is also higher in areas with a high incidence of PTB [3]. And the incidence of PTB has remained stubbornly high in recent years due to the increase of drug-resistant PTB, aggravation of air pollution and decrease of human immunity [3, 4]. Therefore, the incidence of PTB with lung cancer has been on an upward trend.

Because the early aetiology of lung cancer is unknown and both lung cancer and PTB are respiratory and consumption diseases, there are similarities in the clinical and imaging features between the two [5, 6]. These similarities easily lead to missed diagnosis or misdiagnosis and consequently delay treatment, especially for early lung cancer patients with a strongly positive result of tuberculin test and with sputum smear-positive tuberculin [5]. Therefore, early diagnosis with prompt treatment is critical for patients with PTB and lung cancer, and for the control of tumour progression. Comprehensive therapy such as surgery, radiotherapy and chemotherapy on the basis of active anti-tubercular treatment can effectively improve the prognosis of patients. Hence this review and meta-analysis collected the published relevant literature to compare the clinical features and CT imaging features between PTB with lung cancer and PTB alone, and to analyse the differences between the two. This study aims to provide a theoretical basis for the clinical diagnosis and treatment of the patients.

## Methods

The systematic review followed the methodology outlined in Cochrane Handbook for Systematic Reviews of Interventions Version 6.0 [7]. And this review was reported in accordance with Preferred Reporting Items for Systematic Reviews and Meta-Analyses Protocols (PRISMA) [8].

### Search strategy

Case-control studies related to the clinical and CT imaging characteristics between PTB with lung cancer and PTB alone were systematically search from PubMed, Embase, Cochrane, WangFang Data and China National Knowledge Infrastructure up to 6 July 2021, to provide a comprehensive comparison of clinical and imaging features between the two. The search items were as follows: ‘lung cancer’, ‘lung cancer with pulmonary tuberculosis’, ‘pulmonary tuberculosis’, ‘clinical features’ and ‘CT images’. In addition, the references of the preliminarily included articles in the above systematic search were also searched to prevent the omission of any related articles. Based on the literature retrieval, a comprehensive comparison and report on the similarities and differences of clinical and imaging features between the two types of patients had been achieved.

### Inclusion and exclusion criteria

Two researchers independently assessed the titles and abstracts of the articles obtained from the initial search according to the inclusion and exclusion criteria. In case of disagreement during this process, the third researcher would be consulted, who would finally determine whether to include the controversial article or exclude based on the opinion of the former two. Inclusion criteria were: (1) retrospective case-control study; (2) the subjects were PTB patients, and were divided into the study (PTB with lung cancer) and control (PTB along) groups; (3) analysis indexes included clinical symptoms and imaging features of the two groups. Exclusion criteria were: (1) duplicated publication of the same trial; (2) the full text was not available, or the data were incomplete and were not available through reasonable channels; (3) with major deficiencies in study design (such as insufficient data of study subjects and no retrospective analysis of control group) or major biases in the reporting of results. Studies that met any of the criteria were excluded.

### Data extraction and quality assessment

Two researchers independently extracted the following information provided by each included study into a table: title, first author, journal of publication, year of publication, number of included study subjects, grouping, age of study subjects, inclusion criteria and exclusion criteria, diagnostic criteria for PTB and lung cancer, clinical characteristics and imaging characteristics of patients, and study design-related indicators (mainly including study protocol and quality control). After data extraction, a third researcher checked the consistency of the data extracted by the former two researchers.

The quality of the included studies was assessed by two researchers independently using the Newcastle-Ottawa Scale (NOS) for observational case-control studies [9]. The evaluation items include: (1) selection of controls and cases: definition of controls and cases, source of controls and cases; (2) comparability of controls and cases; (3) ascertainment of exposure. The observational study with 6–9 points was considered as high quality, 4 or 5 as medium quality, and 3 or less as low quality. In case of disagreement arising about the results of the quality assessment, a third researcher would participate in the discussion and made a final judgment on the scores based on the opinions of the former two researchers.

### Statistical analysis

Results were merged across studies with STATA version 15.1 (Stata Corp MP., College Station, TX, USA) [10, 11]. Study subjects in each included study were patients with PTB and lung cancer in study group and patients with PTB along with control group, suggesting a good clinical consistency. Assessment of heterogeneity was performed using *Q* test and *I*^2^ statistics. *I*^2^ values of 0–39%, 40–59% and 60–90% indicated low, moderate and high heterogeneity among studies, respectively [7]. In case of low heterogeneity, fixed-effects model was adopted for pooling results; otherwise, random-effects model was employed. For dichotomous variables, odds ratios (ORs) and its 95% confidence interval (CI) were utilised to compare the clinical and imaging characteristics of PTB patients with lung cancer with those of PTB patients alone. If the number of studies for the comparison was ⩾6, the results were presented in forest plots and tables, otherwise in tables. If the number of studies was ⩾6, Egger's test was used to assess the publication bias of the results and Duval and Tweedie’ s trim and fill test for evaluating the sensitivity of the results [12, 13]. *P* < 0.05 suggested a significant difference except that *P* < 0.10 in the results of Egger's test was considered statistically significant.

## Results

### Literature search, study characteristics and quality assessment

**Fig. 1.**
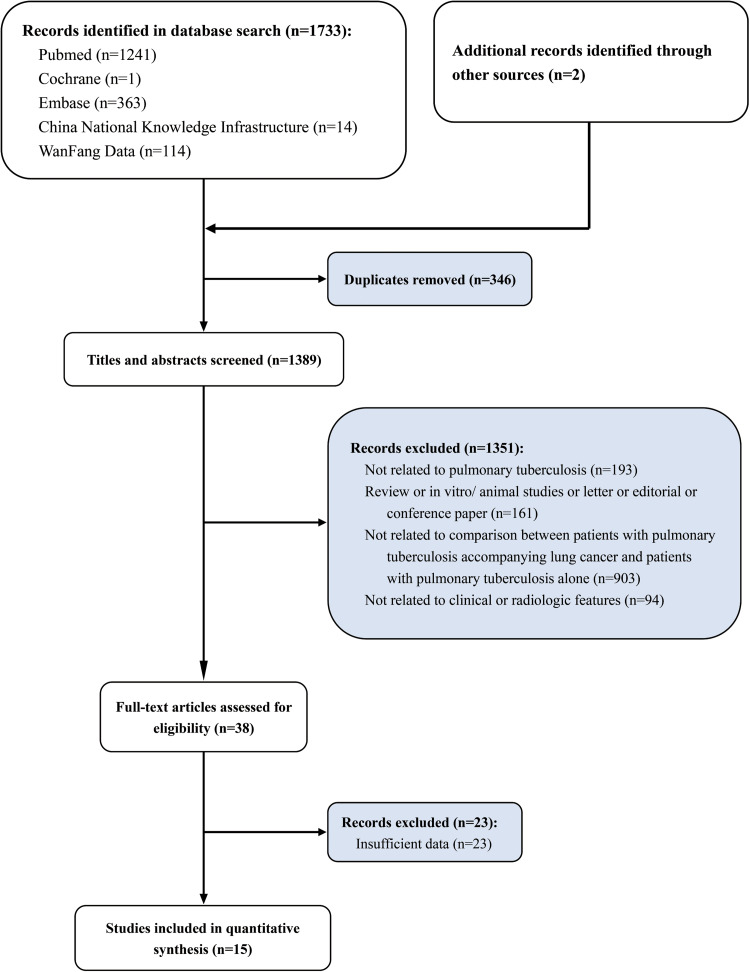
Study selection flowchart, systematic review and meta-analysis of comparison of clinical and imaging features between pulmonary tuberculosis complicated with lung cancer and simple pulmonary tuberculosis.

In total, 1733 articles were obtained by systematic retrieval in five database, and two articles by manual retrieval from the references of the initially included articles. After removal of 346 duplicate articles, the titles and abstracts of the resulting article were screened and 1351 articles that did not meet the inclusion criteria were then excluded (not related to PTB, *n* = 193; review or *in vitro*/animal studies or letter or editorial or conference paper, *n* = 161; not related to comparison between patients with PTB accompanying lung cancer and patients with PTB alone, *n* = 903; not related to clinical features or radiologic characteristic, *n* = 94). Subsequently, by reading full text, 23 articles without valid data were excluded. Finally, 15 studies were included in the meta-analysis (Fig. 1), including 908 patients with PTB and lung cancer and 1151 patients with PTB alone. The basic characteristics of 15 retrospective case-control studies included in the meta-analysis are shown in Table 1 [14–28].

**Table 1.** Baseline characteristics of included studies for meta-analysis

Author	No. of cases		Age (years)	Definition of PTB and LC	NOS score
	PTB and LC	PTB			
Zhang *et al*. [14]	42	42	60.57 ± 10.12	PTB: acid-fast mycobacterium test LC: cytological and histopathological examinations	9
Sun [15]	50	50	66.20 ± 3.04	PTB: acid-fast mycobacterium test LC: cytological and histopathological examinations	5
Wang and Du [16]	40	40	64.12 ± 11.37	PTB: acid-fast mycobacterium test LC: cytological and histopathological examinations	5
Xu *et al*. [17]	80	80	45.38 ± 5.32	PTB: acid-fast mycobacterium test LC: cytological and histopathological examinations	8
Qian *et al*. [18]	47	312	63.9 ± 5.87	PTB: acid-fast mycobacterium test LC: cytological and histopathological examinations	9
Li *et al*. [19]	40	40	47.29 ± 1.63	PTB: acid-fast mycobacterium test LC: cytological and histopathological examinations	6
Shang [20]	60	60	50.40 ± 7.10	PTB: acid-fast mycobacterium test LC: cytological and histopathological examinations	7
Han and Xie [21]	40	40	65.30 ± 2.30	PTB: acid-fast mycobacterium test LC: cytological and histopathological examinations	5
Zhang [22]	50	54	57.10 ± 6.70	PTB: acid-fast mycobacterium test LC: cytological and histopathological examinations	7
Cong *et al*. [23]	45	23	71.50 ± 4.20	PTB: acid-fast mycobacterium test LC: cytological and histopathological examinations	5
Liu *et al*. [24]	118	120	63.50 ± 5.40	PTB: acid-fast mycobacterium test LC: cytological and histopathological examinations	6
Zhao *et al*. [25]	80	80	61.10 ± 7.70	PTB: acid-fast mycobacterium test LC: cytological and histopathological examinations	9
Wang *et al*. [26]	68	60	62.75 ± 8.30	PTB: acid-fast mycobacterium test LC: cytological and histopathological examinations	9
Quan *et al*. [27]	64	60	61.20 ± 15.28	PTB: acid-fast mycobacterium test LC: cytological and histopathological examinations	7
Wang *et al*. [28]	84	90	58.98 ± 6.21	PTB: acid-fast mycobacterium test LC: cytological and histopathological examinations	7

PTB, pulmonary tuberculosis; LC, lung cancer; NOS, Newcastle-Ottawa Scale.

Detail of acid-fast mycobacterium test: acid-fast mycobacterium was detected in sputum smear or in bronchoalveolar lavage fluid or bronchoscope brush.

In terms of quality assessment, all the 15 studies were considered to be of medium or high quality, with scores of 5–9 (Table 1). In addition, before the start of the analysis, patients with the following conditions were excluded from each study: (1) previous history of pulmonary surgery; (2) combined with other types of diseases in the chest, lung and chest wall; (3) combined with mental system abnormalities. Therefore, there was no significant data loss in each study, causing no marked damage to the power of the test, but affecting the extrapolation of the study results. In summary, the overall evaluation of the included studies considered good quality, and high reliability of the meta-analysis results.

### Clinical features

**Fig. 2.**
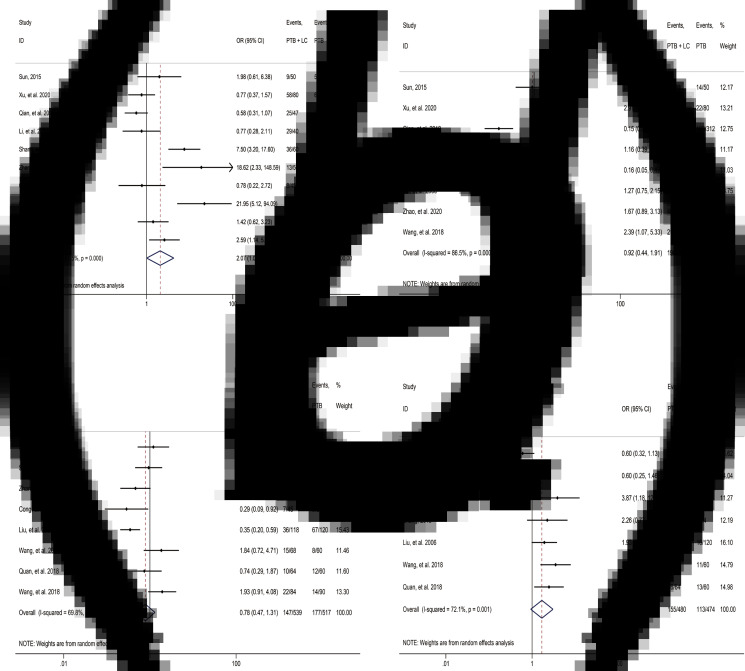
Forest plot of clinical features of comparison between patients with PTB and LC and patients with PTB alone: (a) persistent chest pain; (b) haemoptysis; (c) fever; (d) emaciation.

The meta-analysis results showed no significant difference in most clinical symptoms of the patients in the two groups (persistent chest pain: OR 2.07, 95% CI 1.00–4.27; chest distress: OR 1.62, 95% CI 0.92–2.82; haemoptysis: OR 0.92, 95% CI 0.44–1.91; fever: OR 0.78, 95% CI 0.47–1.31; emaciation: OR 1.69, 95% CI 0.93–3.06; shortness of breath: OR 1.22, 95% CI 0.74–2.03; cervical lymphadenectasis: OR 1.02, 95% CI 0.71–1.46, Table 2, Fig. 2).

**Table 2.** Summarised results of included studies

Indicators	No. of studies	Sample size	Effect size (95%CI)	Model	Heterogeneity (%)	
					*I*^2^	*P*
*Clinical features*						
Persistent chest pain	10	1517	2.07 (1.00–4.27)	Random	82.5	<0.001
Chest distress	2	240	1.62 (0.92–2.82)	Fixed	0.0	1.000
Haemoptysis	8	1379	0.92 (0.44–1.91)	Random	86.5	<0.001
Fever	8	1056	0.78 (0.47–1.31)	Random	69.8	0.002
Emaciation	7	954	1.69 (0.93–3.06)	Random	72.1	0.001
Shortness of breath	2	240	1.22 (0.74–2.03)	Fixed	0.0	1.000
Cervical lymphadenectasis	4	538	1.02 (0.71–1.46)	Fixed	0.0	0.657
Irritable cough	11	1695	2.43 (1.43–4.11)	Random	81.7	<0.001
Nigh sweating	7	970	0.56 (0.36–0.87)	Random	51.4	0.054
Dyspnoea	3	406	3.58 (1.01–12.72)	Random	62.2	0.071
Bloody sputum	5	596	1.46 (1.02–2.10)	Fixed	0.0	0.697
Haemorrhagic pleural effusion	5	921	5.73 (1.63–20.12)	Random	84.6	<0.001
*Radiologic features*						
Calcified shadow	7	982	1.93 (0.51–7.25)	Random	91.9	<0.001
Mass shadow	5	1015	7.26 (0.86–61.03)	Random	96.8	<0.001
Lobulation sign	7	902	7.67 (3.49–16.84)	Random	79.2	<0.001
Mass	3	264	11.94 (5.74–24.84)	Fixed	30.6	0.236
Nodular shadow	5	586	4.92 (2.79–8.66)	Fixed	14.5	0.322
Satellite lesion	4	408	9.29 (2.86–30.18)	Random	75.1	0.007
Small vacuole sign	2	168	5.07 (1.31–19.66)	Random	59.0	0.118
Vacuole sign	3	414	7.86 (3.40–18.16)	Fixed	0.0	0.984
Spicule sign	9	1180	6.66 (4.85–9.16)	Fixed	0.0	0.825
Pleural indentation	5	568	3.18 (2.14–4.73)	Fixed	0.0	0.561
Cord-like shadow	5	598	0.21 (0.13–0.33)	Fixed	0.0	0.954
Cavitary lesions	12	1767	0.30 (0.23–0.38)	Fixed	0.0	0.981

**Fig. 3.**
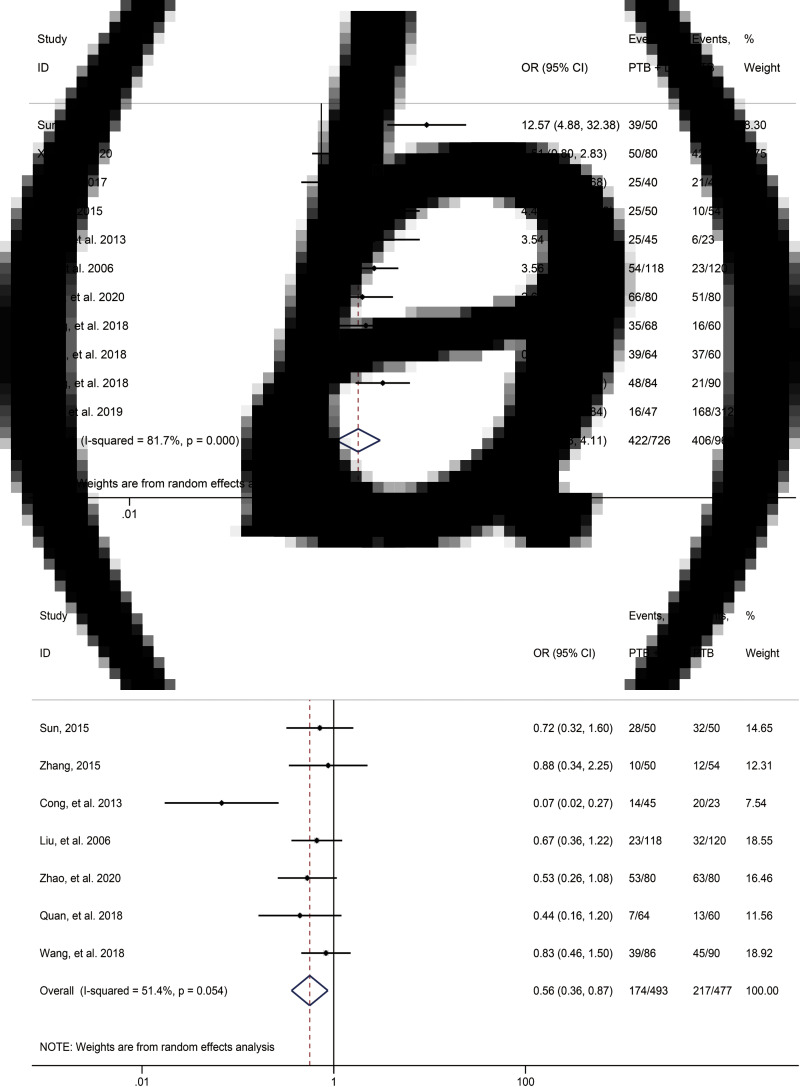
Forest plot of clinical features of comparison between patients with PTB and LC and patients with PTB alone: (a) irritable cough; (b) night sweating.

However, the meta-analysis results also summarised some clinical characteristics to distinguish the two types of patients. Specifically, by using random-effects model, irritable cough was found to be of a significantly higher frequency in patients with PTB and lung cancer in comparison with that in patients with PTB alone (OR 2.43, 95% CI 1.43–4.11; Table 2, Fig. 3a). In terms of night sweating (reported in seven articles), patients with PTB and lung cancer had a significantly lower proportion of this symptom (OR 0.56, 95% CI 0.36–0.87; Table 2, Fig. 3b). Additionally, compared with patients with PTB alone, patients with PTB and lung cancer were associated with higher frequencies of dyspnoea (OR 3.58, 95% CI 1.01–12.72; Table 2), bloody sputum (OR 1.46, 95% CI 1.02–2.10; Table 2) and haemorrhagic pleural effusion (OR 5.73, 95% CI 1.63–20.12; Table 2).

### Radiologic features

**Fig. 4.**
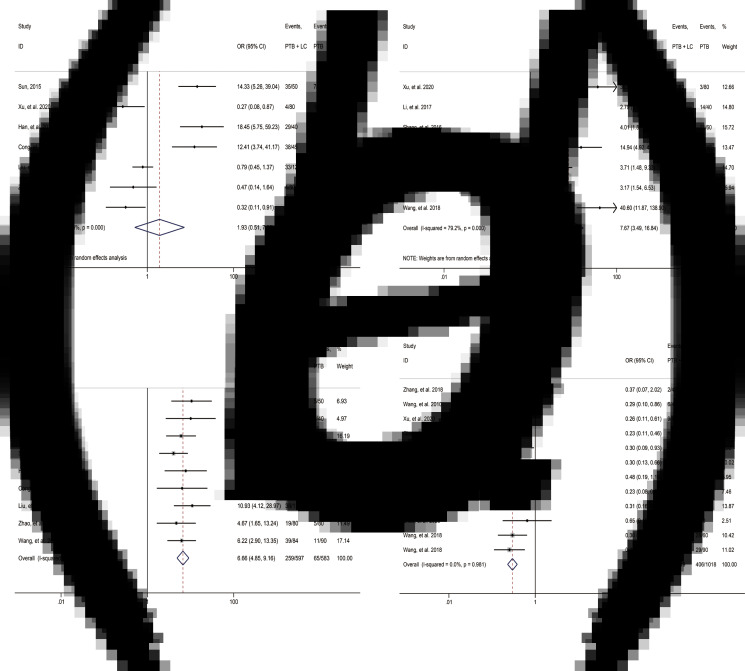
Forest plot of radiologic features of comparison between patients with PTB and LC and patients with PTB alone: (a) calcified shadow; (b) lobulation sign; (c) spicule sign; (d) cavitary lesions.

Imaging characteristics between the two types of patients were also summarised and compared to obtain the similarities and differences. No significant difference was identified in calcified shadow (OR 1.93, 95% CI 0.51–7.25; Table 2, Fig. 4a) and mass shadow (OR 7.26, 95% CI 0.86–61.03; Table 2) between the two groups. However, among the imaging signs, patients with PTB and lung cancer showed a higher proportion of lobulation sign, mass, nodular shadow, satellite lesion, small vacuole sign, vacuole sign, spicule sign and pleural indentation compared with the imaging features of patients with PTB alone (lobulation sign: OR 7.67, 95% CI 3.49–16.84; mass: OR 11.94, 95% CI 5.74–24.84; nodular shadow: OR 4.92, 95% CI 2.79–8.66; satellite lesion: OR 9.29, 95% CI 2.86–30.18; small vacuole sign: OR 5.07, 95% CI 1.31–19.66; vacuole sign: OR 7.86, 95% CI 3.40–18.16; spicule sign: OR 6.66, 95% CI 4.85–9.16; pleural indentation: OR 3.18, 95% CI 2.14–4.73; Table 2, Fig. 4b and c). And patients with PTB and lung cancer had lower proportion of cord-like shadow and cavitary lesions (cord-like shadow: OR 0.21, 95% CI 0.13–0.33; cavitary lesions: OR 0.30, 95% CI 0.23–0.38; Table 2, Fig. 4d).

### Publication bias assessment and sensitivity analysis

We used Egger's test to analyse the publication bias of each indicator. The test result found publication bias in lobulation sign. Duval and Tweedie's trim and fill test proved the stability and guiding significance of the effect size of each index (Table 3).

**Table 3.** Evaluation of publication bias and sensitivity analysis

Index	Egger's regression		Duval and Tweedie's trim and fill		
	Intercept	*P*	Original effect size	Studies trimmed	Adjusted effect size
*Comparison between patients with PTB and LC and patients with PTB alone*					
Persistent chest pain	4.376	0.172	2.07 (1.00–4.27)	0	2.07 (1.00–4.27)
Haemoptysis	−4.398	0.312	0.92 (0.44–1.91)	1	0.77 (0.36–1.63)
Fever	2.527	0.430	0.78 (0.47–1.31)	0	0.78 (0.47–1.31)
Emaciation	4.065	0.284	1.69 (0.93–3.06)	0	1.69 (0.93–3.06)
Irritable cough	4.133	0.320	2.43 (1.43–4.11)	0	2.43 (1.43–4.11)
Night sweating	−3.890	0.159	0.56 (0.36–0.87)	2	0.45 (0.29–0.69)
Calcified shadow	3.158	0.514	1.93 (0.51–7.25)	1	1.34 (0.36–4.92)
Lobulation sign	9.003	0.006	7.67 (3.49–16.84)	0	7.67 (3.49–16.84)
Spicule sign	1.792	0.145	6.66 (4.85–9.16)	3	5.52 (4.15–7.34)
Cavitary lesions	1.030	0.163	0.30 (0.23–0.38)	0	0.30 (0.23–0.39)

PTB, pulmonary tuberculosis; LC, lung cancer.

## Discussion

In quantitative analysis, the high heterogeneity obtained by meta-analysis and the existing publication bias have no serious impact on the comparison result of clinical and imaging characteristics in the two groups of patients. That means that this meta-analysis truly reflects the possible situation in the process of clinical examination and treatment for those two types of patients. First of all, for high heterogeneity and publication bias in lobulation sign, the significance of its effect sizes is questionable. However, the meta-analysis of this indicator showed good consistency, that is, patients with PTB with lung cancer have a higher proportion of lobulation sign than patients with PTB alone (Table 2, Fig. 4b). And Duval and Tweedie's trim and fill test further proved the stability of meta-analysis results (Table 3). Therefore, we believe that high heterogeneity may be related to individual variation of patients in the study design, and follow-up studies required by publication bias may not have a significant effect on the stability of the results. Similarly, the high heterogeneity in irritable cough and night sweating also reflects the individual variation that may occur during clinical examination and treatment of these two types of patients (Table 2, Fig. 3a and b). Collectively, based on the evidence of quantitative analysis, patients with lung cancer and PTB have a higher proportion of irritable cough but less night sweating than patients with PTB alone.

The imaging findings summary confirmed that CT imaging is effective in distinguishing patients with PTB and lung cancer from patients with PTB alone. There are significant differences in the imaging features between the two groups. Specifically, compared with the other group, patients with PTB and lung cancer showed a higher proportion of lobulation sign, mass, nodular shadow, satellite lesion, small vacuole sign, vacuole sign, spicule sign and pleural indentation, but a lower proportion of cord-like shadow and cavitary lesions. And good consistency of each index was observed (Table 2, Fig. 4). This review suggests that in clinical examination and treatment for patients with old PTB in the future, we should pay close attention to the clinical symptoms and follow-up the imaging features of the patients, thus timely identifying whether the patients are accompanied by lung cancer and consequently prompt treatment for improving patients' prognosis.

PTB is one of the most common risk factors of lung cancer. Compared with the general population, PTB patients have a 50% increased risk of lung cancer. The presence of potential TB is significantly related to the increased risk of lung cancer. Especially in patients with more than 20 years of history of PTB, the risk of developing lung cancer is more than 2.5 times higher than that of the general population [29, 30]. The pathogenesis of PTB complicated with lung cancer may be due to the following reasons. First, in response to TB inflammation, the columnar epithelium of tuberculous cavity wall or the columnar epithelium of the cystic dilated bronchial wall shows proliferative changes, squamous metaplasia and consequently carcinogenesis [31]. Second, abnormal cellular immune function in PTB patients, such as the imbalance of Thl/Th2 proportion in helper T cells or the decrease of CD4^+^ T cells, will lead to the weakening of anti-tumour immune function and the increase of cancer cell escape, thus promoting tumour formation [31]. In addition, the combination of chemotherapy and anti-PTB drugs will inhibit the proliferation of human immune lymphocytes and macrophages, thereby blocking antibody formation but promoting carcinogenesis. Third, the calcified foci and calcified lymph nodes after healing of TB can act as a local mechanical irritant to stimulate the adjacent bronchi and cause carcinogenesis [31].

In summary, when PTB patients, especially those with a history of more than 20 years, suddenly experience symptoms such as irritable cough and haemorrhagic pleural effusion after regular anti-PTB treatment, attention should be paid to whether they have lung cancer in addition to PTB recurrence. At the same time, it is necessary to combine the imaging features and laboratory results to accurately diagnose and treat the patients, aiming to improve the survival rate and prognosis of those with PTB and lung cancer.

This review still has some limitations. First, only retrospective case-control studies are included in this review, which can't confirm the direct correlation of clinical symptoms and imaging features with PTB complicated with lung cancer, but only play a suggestive role. Second, the meta-analysis results of some indexes have high heterogeneity, so their guiding significance for clinical diagnosis and treatment is difficult to clarify.

In conclusion, the meta-analysis summarises the similarities and differences in clinical symptoms and imaging characteristics between patients with PTB and lung cancer and patients with PTB alone, suggesting that we should be alert to the occurrence of lung cancer in patients with old PTB recurrence. Specifically, we should pay close attention to the clinical symptoms and follow-up the imaging features of the patients, thus timely identifying whether the patients are accompanied by lung cancer and consequently prompt treatment for improving patients' prognosis.

## Data Availability

The datasets used and/or analysed during the current study are available from the corresponding author on reasonable request.
